# Ionosphere-Constrained Triple-Frequency Cycle Slip Fixing Method for the Rapid Re-Initialization of PPP

**DOI:** 10.3390/s19010117

**Published:** 2018-12-31

**Authors:** Fuxin Yang, Lin Zhao, Liang Li, Jianhua Cheng, Jie Zhang

**Affiliations:** College of Automation, Harbin Engineering University, Harbin 150001, China; yangfuxin@hrbeu.edu.cn (F.Y.); zhaolin@hrbeu.edu.cn (L.Z.); chengjianhua@hrbeu.edu.cn (J.C.); jiezhang@hrbeu.edu.cn (J.Z.)

**Keywords:** precise point positioning (PPP), triple-frequency, cycle slip fixing, ionospheric-constrained, re-initialization

## Abstract

The re-initialization of precise point positioning (PPP) can be avoided by cycle slip detection and correction. Ionospheric delay is critical for cycle slip detection and correction, especially for a long data gap. The frequency diversity from GNSS modernization provides the potential for mitigating the impact of ionospheric delay on cycle slip detection and correction. The proposed method constructs the extra-wide lane (EWL), the wide lane (WL), and the narrow lane (NL) epoch-differenced linear combinations based on the ionosphere constrain criterion, so as to determine the undifferenced cycle slips from the cascading ambiguity resolution. The experiment results show that the cycle slips can be fixed correctly even though cycle slips occur in all the available carrier phase observations, and the 3 min data gaps can be merged without high precision positioning continuity loss. The kinematic experiment shows that the instantaneous re-initialization can be achieved with the proposed method.

## 1. Introduction

The precise point positioning (PPP) technique utilizes undifferenced carrier phase and code observations and applies precise satellite orbit and clock corrections to perform high-precision positioning at a single Global Navigation Satellite System (GNSS) receiver [1]. It is normally recognized that PPP is free from the constraint of baseline lengths, which can be a cost effective technique for real-time navigational applications [2]. However, the PPP requires a relatively long initialization period, few tens of minutes at least, for the phase ambiguity to converge [3]. When loss-of-lock in signal tracking occurs, the carrier phase observations often suffer from cycle slips, and the re-initialization of PPP which needs tens of minutes to get back the desired accuracy will be required.

The conventional PPP implementations typically re-initialize the ambiguity estimation when the cycle slips are detected [4]. This processing does not exploit the integer nature of cycle slips and can result in frequent re-initializations, especially for the case that all carrier phase observations simultaneously suffer from cycle slips. Cycles slips often occur during magnetic storms [5] and under scintillations [6]. Moreover, a moving receiver increases the risk of experiencing cycle slips on one or more satellites [4]. Therefore, in order to determine the integer cycle slips between epochs and avoid the re-initialization of PPP, a number of cycle slip detection and correction methods have been developed and implemented, which can be classified into three groups.

The first group is the observation time series analysis. One typical time series analysis based example is the TurboEdit method, which makes use of the Melborne–Wubbena (MW) linear combination together with ionospheric combination [7]. A few modifications to the TurboEdit method have been studied to improve the performance of cycle slip detection and correction under high sampling rate observation and high ionospheric activity [8,9]. However, it requires several minutes of continuous phase observation to implement cycle slip correction. Therefore, they are not suitable for the rapid re-initialization of PPP.

The second group utilizes multi-frequency observations linear combination and implements cycle slip detection and correction separately. With the rapid development of GNSS modernization, the new generations of GNSS provides three or more frequencies signals [10]. The frequency diversity provides more observation combinations, which will benefit for cycle slip detection and correction. Dai et al. employed two geometry-free (GF) combinations of triple-frequency carrier phase observations to detect and correct cycle slips under high data rate [11]. De Lacy et al. defined more than five types of linear combinations of triple-frequency observations to detect and correct cycle slips [12]. Zhao et al. presented a cycle slip detection and correction method based on independent linear combinations of undifferenced triple-frequency observations [13]. Huang et al. employed two GF carrier phase combinations and one GF code minus phase linear combination to detect and correct cycle slip [14]. A common feature of the above-mentioned combination methods is based on assumption that the change of ionospheric delay between epochs can be ignored. However, the validation of assumption is challenged by the fact that the temporal ionospheric delay will increase when the data gap enlarges. Therefore, it can be anticipated that the success rate of cycle slip detection and correction can be improved by mitigating the ionospheric delay in case of long data gap or ionospheric scintillation.

In the third group, the cycle slip detection and correction is completed by the resolution of the epoch-differenced ambiguity, which is widely used for the rapid re-initialization of PPP. The epoch-differenced cycle slip fixing involves three steps, i.e., detecting, estimating and correcting the cycle slips [15]. The dual-frequency epoch-differenced cycle slip fixing method has been investigated by Banville and Langley, and Zhang and Li [16,17]. Zhang and Li extended this method to triple-frequency observations to obtain a better performance of cycle slip fixing [18]. Although the temporal ionospheric delay between epochs is well considered, the advantages of multi-frequency observation combination on ionospheric mitigation have not been exploited to improve the reliability of cycle slip fixing. Moreover, triple-frequency cycle slips fixing method with a long data gap has not been investigated.

The availability of multi-frequency observations introduces more degrees of freedom in the ionosphere mitigation combination. In order to mitigate the impact of ionosperic delay, we proposed an ionosphere-constrained triple-frequency cascading cycle slip fixing method to obtain the rapid re-initialization of PPP. Firstly, we introduce the observation model and error handling strategy. Next, we detailed the linear combination for cascading EWL-WL-NL cycle slip fixing under the criterion of ionosphere-constrain. In the experiment section, the performance of proposed method has firstly been tested by using static data, with the injected frequent cycle slips into triple-frequency carrier phase observations. Then, the real kinematic data has also been used to assess the rapid re-initialization performance of PPP. Finally, some conclusions and remarks are summarized, and an outlook for future research is presented.

## 2. Methodology

### 2.1. Observation Model and Error Handling

The linear raw observations on frequency *g* (*g* = 1, 2, 3) from rover to satellite *j* (*j* = 1, … s) can be modeled as [19],

(1) $$L_{g}^{j}=\rho^{j}+dt_{r}-dt^{j}-\gamma_{g}\cdot I_{1}^{j}+T^{j}+\lambda_{g}\cdot (\omega_{g}^{j}+N_{g}^{j})+\lambda_{g}\cdot (b_{g}-b_{g}^{j})+\xi_{g}^{j}$$

(2) $$P_{g}^{j}=\rho_{j}+dt_{r}-dt^{j}+\gamma_{g}\cdot I_{1}^{j}+T^{j}+d_{g}-d_{g}^{j}+\varepsilon_{g}^{j}$$

where *L* and *P* denote carrier phase and code observations, respectively; $\rho^{j}$ denotes the geometric distance between satellite and rover; $dt_{r}$ and $dt^{j}$ are the clock biases of receiver and satellite, respectively; $I_{1}^{j}$ is the slant ionosphere refraction on $f_{1}$, $\gamma_{g}=f_{1}^{2}/f_{g}^{2}$ is the ionospheric factor of $f_{g}$ with respect to $f_{1}$; $T^{j}$ is slant tropospheric delay; $\lambda_{g}$ is the carrier phase wavelength at $f_{g}$; $\omega_{g}^{j}$ is the phase wind up delay; $N_{g}^{j}$ is the integer phase ambiguity at $f_{g}$; $d_{g}$ and $d_{g}^{j}$ are the code biases for receiver and satellite, respectively; $b_{g}$ and $b_{g}^{j}$ are the uncalibrated phase delays (UPDs) for receiver and satellite, respectively. $\varepsilon_{g}^{j}$ and $\xi_{g}^{j}$ are the sum of multipath effects and thermal noise for the code and carrier phase observations, respectively. Noted that all variables are expressed in meters, expect the ambiguity, UPDs, and the phase wind-up delay in cycles.

The observation errors presented in (1) and (2) have to be corrected, because the ambiguity validation for cycle slip fixing is not reliable in the presence of biases [20]. The satellite orbit and clock can be corrected using external precise products [18,21]. The effects of phase wind up, phase center offsets (PCO), and phase center variations (PCV) at satellite and receiver antenna, as well as the site displacement effects, can be corrected using empirical models [17,22]. The epoch-differenced model will eliminate the slowly varying parameters, such as code instrumental biases, UPDs, and tropospheric wet component. For brevity, the epoch-differenced model by omitting the noise and multipath items is formulated as,

(3) $$\Delta P_{g}^{j}=\Delta {\bar{\rho }}_{j}+\gamma_{g}\cdot \Delta I_{1}^{j}$$

(4) $$\Delta L_{g}^{j}=\Delta {\bar{\rho }}_{j}-\gamma_{g}\cdot \Delta I_{1}^{j}+\lambda_{g}\cdot \Delta N_{g}^{j}$$

where Δ denotes the difference between two consecutive epochs. $\Delta P_{g}^{j}$ and $\Delta L_{g}^{j}$ refer to the epoch-differenced code and phase observations corrected by the above-mentioned errors. $\Delta {\bar{\rho }}_{j}$ refers to the change of the non-dispersive delay including the geometric and receiver clock error. $\Delta I_{1}^{j}$ is the slant ionospheric delay variation on $f_{1}$ of satellite *j*. $\Delta N_{g}^{j}$ denotes the integer cycle slip, which is zero unless a cycle slip occurs. It can be found that the ionospheric variation and the omitting combined observation noise are two crucial factors affecting the reliability of the epoch-differenced cycle slip fixing.

### 2.2. EWL-WL-NL Cascading Cycle Slip Fixing

Through a linear combination of triple-frequency observations, the cascading cycle slip fixing method is proposed based on the independent EWL, WL, and NL linear combinations. Meanwhile, in order to ensure cycle slip fixing correctly and reliably, the ionospheric variation and the ratio of noise to the wavelength will be focused at each step.

On the basis of raw triple-frequency observations, the linearly combined carrier phase observation can be defined as [23]

(5) $$L_{\left(l,m,n\right)}=\frac{l\cdot f_{1}\cdot L_{1}+m\cdot f_{2}\cdot L_{2}+n\cdot f_{3}\cdot L_{3}}{l\cdot f_{1}+m\cdot f_{2}+n\cdot f_{3}}$$

where $L_{\left(l,m,n\right)}$ is the linearly combined carrier phase observation, and the combination coefficients *l*, *m*, and *n* are arbitrary integers.

The linearly combined code observation, wavelength, and integer ambiguity are respectively defined as,

(6) $$P_{\left(l,m,n\right)}=\frac{l\cdot f_{1}\cdot P_{1}+m\cdot f_{2}\cdot P_{2}+n\cdot f_{3}\cdot P_{3}}{l\cdot f_{1}+m\cdot f_{2}+n\cdot f_{3}}$$

(7) $$\lambda_{\left(l,m,n\right)}=\frac{c}{l\cdot f_{1}+m\cdot f_{2}+n\cdot f_{3}}$$

(8) $$N_{\left(l,m,n\right)}=l\cdot N_{1}+m\cdot N_{2}+n\cdot N_{3}$$

Taking BDS as an example, the triple-frequency BDS signals centered at B1 (1561.098 MHz), B2 (1207.14 MHz), and B3 (1268.52 MHz). We assume that the standard deviation (STD) of BDS code and carrier phase noises on three frequencies are equal to each other, i.e., $\sigma_{P_{1}}=\sigma_{P_{2}}=\sigma_{P_{3}}=0.3\;m$, $\sigma_{L_{1}}=\sigma_{L_{2}}=\sigma_{L_{3}}=0.003\;m$ for the numerical analysis, which will be convenient to show the edge of our proposed method.

#### 2.2.1. EWL Cycle Slip Fixing

The EWL cycle slip is determined by the Hatch–Melbourne–Wübbena (HMW) combination in adjacent epochs between *k* and *k* + 1 [24,25,26]. The epoch-differenced HMW combination eliminates the geometry and frequency dependent errors, which can be expressed as,

(9) $$\Delta L_{EWL}^{j}=\Delta L_{\left(0,1,-1\right)}^{j}-\Delta P_{\left(0,1,1\right)}^{j}=\lambda_{\left(0,1,-1\right)}\cdot \Delta N_{\left(0,1,-1\right)}^{j}$$

where

(10) $$\{\begin{array}{c} \lambda_{\left(0,1,-1\right)}\approx 4.884m\\ \sigma_{\Delta L_{EWL}^{j}}\approx \sigma_{P_{1}} \end{array}$$

in which $\lambda_{\left(0,1,-1\right)}$ is the wavelength for EWL, $\Delta N_{\left(0,1,-1\right)}$ is the EWL cycle slip in cycles, and $\sigma_{\Delta L_{EWL}^{j}}$ denotes the STD of $\Delta L_{EWL}^{j}$. For brevity, the combined multipath and noise are not given in observations combination. The EWL cycle slip is determined by rounding as follows,

(11) $$\Delta N_{\left(0,1,-1\right)}^{j}=round(\Delta L_{EWL}^{j}/\lambda_{\left(0,1,-1\right)})$$

where *round* makes the element to the nearest integer. Positive element with a fractional part of 0.5 round up to the nearest positive integer, and negative element with a fractional part of −0.5 round down to the nearest negative integer.

The proposed EWL cycle slip fixing is ionosphere-free through the HMW combination. It can be found that the ratio of noise to the wavelength is 0.06 cycles, which indicates the success rate of EWL cycle slip fixing using the constructed HMW combination is close to 100%.

#### 2.2.2. WL Cycle Slip Fixing

The WL cycle slip fixing is firstly carried out with both the WL and the EWL carrier-phase observables in adjacent epochs between *k* and *k* + 1 for satellite *j*,

(12) $$\{\begin{array}{c} \Delta L_{\left(1,-1,0\right)}^{j}=\Delta {\bar{\rho }}_{j}-\frac{f_{1}}{f_{2}}\cdot \Delta I_{1}^{j}+\lambda_{\left(1,-1,0\right)}\cdot \Delta N_{\left(1,-1,0\right)}^{j}\\ \Delta L_{\left(0,1,-1\right)}^{j}-\lambda_{\left(0,1,-1\right)}\cdot \Delta N_{\left(0,1,-1\right)}^{j}=\Delta {\bar{\rho }}_{j}-\frac{f_{1}^{2}}{f_{2}f_{3}}\cdot \Delta I_{1}^{j} \end{array}$$

where $\Delta L_{\left(1,-1,0\right)}$ and $\Delta L_{\left(0,1,-1\right)}$ are epoch-differenced WL and EWL carrier phase observations, respectively.

In order to mitigate the ionospheric delay, we can form an ionosphere-free (IF) phase combination and use the IF code combination based on (13) to assist in WL cycle slip fixing,

(13) $$\{\begin{array}{c} \Delta P_{3}^{j}=\frac{f_{1}^{2}}{f_{1}^{2}-f_{2}^{2}}\Delta P_{1}^{j}-\frac{f_{2}^{2}}{f_{1}^{2}-f_{2}^{2}}\Delta P_{2}^{j}=\Delta {\bar{\rho }}^{j}\\ \Delta L_{WL}^{j}=\frac{f_{1}}{f_{1}-f_{3}}\Delta L_{\left(1,-1,0\right)}^{i}-\frac{f_{3}}{f_{1}-f_{3}}(\Delta L_{\left(0,1,-1\right)}^{j}-\lambda_{\left(0,1,-1\right)}\cdot \Delta N_{\left(0,1,-1\right)}^{j})=\Delta {\bar{\rho }}^{j}+\lambda_{\left(1,-1,0\right)}\cdot \frac{f_{1}}{f_{1}-f_{3}}\cdot \Delta N_{\left(1,-1,0\right)}^{j} \end{array}$$

where

(14) $$\{\begin{array}{c} \frac{f_{1}}{f_{1}-f_{3}}\cdot \lambda_{\left(1,-1,0\right)}\approx 5.33\times 0.847\;m\\ \sigma_{\Delta L_{WL}}\approx 179.878\cdot \sigma_{L_{1}} \end{array}$$

where $\Delta P_{3}$ and $\Delta L_{WL}$ are the epoch-differenced code and the WL carrier phase observations based on IF combination, respectively. $\Delta N_{\left(1,-1,0\right)}^{j}$ denotes the cycle slip on WL combination. In accordance with the variance-covariance propagation law, although the STD of $\Delta L_{WL}$ is amplified by about 180 times to 0.54 m, the WL wavelength is amplified by 5.33 times to about 4.51 m. Assuming that there is no bias in the WL IF combination, the corresponding ratio of noise to the wavelength is about 0.12 cycles. Therefore, the WL cycle slip $\Delta N_{\left(1,-1,0\right)}^{j}$ can be reliably fixed using the least-squares ambiguity decorrelation adjustment (LAMBDA) algorithm [27].

#### 2.2.3. NL Cycle Slip Fixing

Compared with the EWL and WL cycle slip fixing, the NL cycle slip fixing with shorter wavelength is more sensitive to the presence biases of epoch-differenced model. Therefore, it is very necessary to restrict the ionospheric variation and the combined observation noise simultaneously for improving the reliability of NL cycle slip fixing.

The NL combination can be constructed to restrict the combined noise as,

(15) $$\{\begin{array}{c} \Delta L_{1}^{j}=\Delta {\bar{\rho }}_{}^{j}-\gamma_{1}\cdot \Delta I_{1}^{j}+\lambda_{\left(1,0,0\right)}\Delta N_{\left(1,0,0\right)}^{j}\\ \Delta L_{2}^{j}+\lambda_{\left(0,1,0\right)}\Delta N_{\left(1,-1,0\right)}^{j}=\Delta {\bar{\rho }}_{}^{j}-\gamma_{2}\cdot \Delta I_{1}^{j}+\lambda_{\left(0,1,0\right)}\Delta N_{\left(1,0,0\right)}^{j}\\ \Delta L_{3}^{j}+\lambda_{\left(0,0,1\right)}(\Delta N_{\left(1,-1,0\right)}^{j}+\Delta N_{\left(0,1,-1\right)}^{j})=\Delta {\bar{\rho }}_{}^{j}-\gamma_{3}\cdot \Delta I_{1}^{j}+\lambda_{\left(0,0,1\right)}\Delta N_{\left(1,0,0\right)}^{j} \end{array}$$

where

(16) $$\{\begin{array}{c} \lambda_{\left(1,0,0\right)}\approx 0.192\;m\\ \lambda_{\left(0,1,0\right)}\approx 0.248m\\ \lambda_{\left(0,1,0\right)}\approx 0.236m\\ \sigma_{\Delta L_{1}}=\sigma_{\Delta L_{2}}=\sigma_{\Delta L_{3}}\approx 1.414\cdot \sigma_{L_{1}} \end{array}$$

However, the ionospheric variation is still in the NL combination, which will affect the reliability of the epoch-differenced cycle slip fixing.

In order to confirm the ionospheric delay variation in (15) and improve the observation redundancy for the NL cycle slip fixing, the temporally relative ionospheric model (TRIM) is established by linear bias model based on a sliding window for modeling and prediction using the random walk process in Kalman filtering [28]. Meanwhile, the temporal correlation for ionospheric delay is demonstrated to be a few minutes [17,29]. Using the triple-frequency observations, the ionospheric observations at *k*th epoch for constructing the TRIM are defined as,

(17) $$\{\begin{array}{c} L_{12}^{j}(k)=L_{1}^{j}(k)-L_{2}^{j}(k)=\lambda_{1}\cdot N_{1}^{j}(k)-\lambda_{2}\cdot N_{2}^{j}(k)+(1-\frac{f_{1}^{2}}{f_{2}^{2}})I_{1}^{j}(k)\\ L_{13}^{j}(k)=L_{1}^{j}(k)-L_{3}^{j}(k)=\lambda_{1}\cdot N_{1}^{j}(k)-\lambda_{2}\cdot N_{3}^{j}(k)+(1-\frac{f_{1}^{2}}{f_{3}^{2}})I_{1}^{j}(k) \end{array}$$

where $L_{12}$ and $L_{13}$ are the frequency-differenced observations. It can be found that the ambiguity is an issue to obtain the undifferenced ionospheric delay. Assuming that the satellite *j* is continuously tracked from the epoch *k* − *n* to *k*, and the ambiguity term is constant during this period, the ionospheric variation among these epochs can be calculated as,

(18) $$\{\begin{array}{c} L_{12}^{j}(k,k-n)=L_{12}^{j}(k)-L_{12}^{j}(k-n)=(1-\frac{f_{1}^{2}}{f_{2}^{2}})\cdot I_{1}^{j}(k,k-n)\\ L_{13}^{j}(k,k-n)=L_{13}^{j}(k)-L_{13}^{j}(k-n)=(1-\frac{f_{1}^{2}}{f_{3}^{2}})\cdot I_{1}^{j}(k,k-n) \end{array}$$

where the ambiguity term is absent and $I_{1}(k,k-n)$ is the ionospheric variation between the (*k* − *n*)th and *k*th epoch at B1. The sliding time window length of *n*, e.g., 2~5 min, is typical value for ionospheric delay variation estimation [17,29]. The estimation values from TRIM can be used to predict the ionospheric variation between the (*k* − *n*)th and *k*th epoch. Therefore, $\Delta I_{1}$ in (15) between the (*k* − *n*)th and *k*th epoch can be calculated by the difference between $I_{1}(k,k-n)$ and $I_{1}(k-1,k-n)$.

The ionospheric variation is mitigated by the TRIM, and the TRIM can usually be quantified with a relatively high confidence level [29]. Assuming that there are no biases in the NL combination, the ratios of noise to the wavelength are $\sigma_{\Delta L_{1}}/\lambda_{\left(1,0,0\right)}\approx 0.022$ cycles, $\sigma_{\Delta L_{2}}/\lambda_{\left(0,1,0\right)}\approx 0.017$ cycles and $\Delta_{L_{3}}/\lambda_{\left(0,0,1\right)}\approx 0.018$ cycles, respectively. Assuming that there is no bias in the constructed NL observations, the NL cycle slip can be fixed reliably using LAMBDA.

According to the description of the proposed method above, the advantage of triple-frequency cascading EWL-WL-NL cycle slip fixing focus on ionosphere mitigation at each step. From (10) for EWL combination, (14) for WL combination and (16) for NL combination, it can be seen that each combination restricts the ratio of the noise to the wavelength within 0.12 cycles after ionospheric delay mitigation. Furthermore, the strength of NL combination is also enhanced by the combined noise restricted and sufficient observation redundancy, which is beneficial for improving the reliability of cycle slip fixing.

To summarize, the implementation of the proposed EWL-WL-NL cascading cycle slip fixing method are as following. Firstly, the HMW combination is constructed for the EWL cycle slip fixing using the integer rounding, and the success rate is up to 100%. When the EWL cycle slip is fixed, the WL cycle slips can be reliably fixed using LAMBDA based on the IF combination. The NL cycle slips can be reliably and correctly fixed through suppressing the ionospheric delay based on the TRIM and the combined observation noise of the epoch-differenced triple-frequency carrier phase observations. Finally, when the EWL, the WL and the NL cycle slips are fixed, the cycle slip on each frequency can be determined.

## 3. Results

In order to evaluate the rapid re-initialization performance of PPP with the proposed method, both the simulated kinematic based on static data and the real-world kinematic data were collected to sufficiently test the proposed cycle slip fixing method. It is noted that the BDS precise orbit and clock products are from the multi-GNSS experiment (MGEX) [30] to avoid the negative effect of the unstable quality of the real-time precise products on the cycle slip fixing because the focus of this paper is the ionospheric delay. Although multi-epoch processing can suppress multipath and provide more redundancy for cycle slip fixing, only adjacent epochs are used in the experiments in order to test the performance under the extreme conditions. The threshold of the ratio test for the LAMBDA algorithm is set as 1/3 for the reliability check of the WL and the NL cycle slip fixing [17,21]. The smoothing window length is set up to 5 min to establish TRIM.

### 3.1. Simulated Kinematic Experiment

In this simulated kinematic experiment, we use observations with 1 s sampling interval from the IGS station JFNG (Trimble NetR9 receiver) in DOY 264, 2016. The time length for simulated kinematic experiment data is 3.5 h. The *Kp* index for the ionosphere activity level is 4 [31], which means a moderate ionosphere activity during the test. The IGS station is selected because the coordinates of JFNG are accurately known. In order to verify the correctness of cycle slips fixing, the cycle slips are removed from the observations through the classical TurboEdit method in the post-processing. The kinematic PPP mode is implemented without constraining the coordinate parameters.

**Figure 1:**
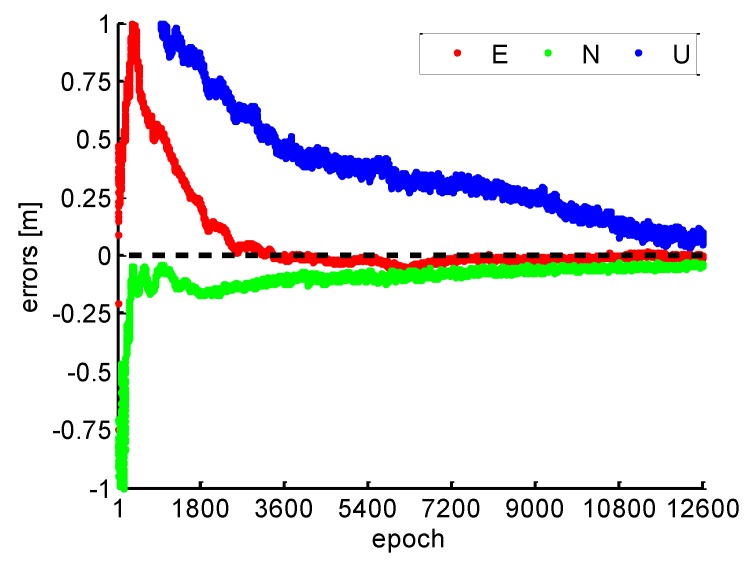
Positioning errors of conventional kinematic precise point positioning (PPP).

As shown in Figure 1, the position errors of east and north components are better than 10 cm after 5400 epoch, and the vertical component has longer convergence and worse position errors than the east and the north. E, N, and U denote the position errors of east, north, and vertical components in Figure 1, respectively. In such case, a long convergence time of PPP is usually required when the re-initialization occurs.

**Figure 2:**
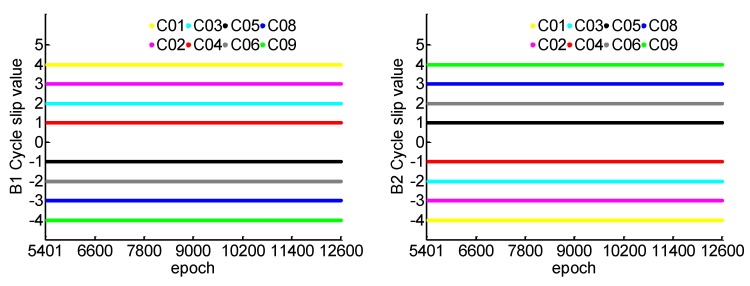

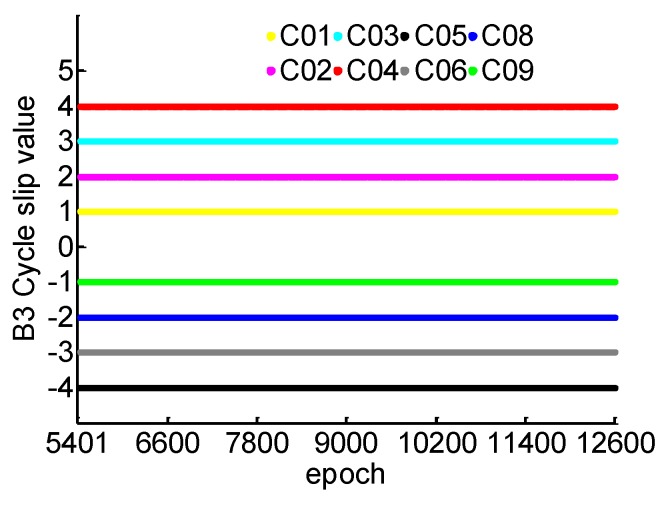
Introduced cycle slip on B1, B2, and B3.

In order to sufficiently test the proposed method, we focus on the worst case of cycle slips, i.e., the carrier phase observations on three frequencies are affected by cycle slips. The simulated cycle slips are generated and injected into the carrier phase observations at every epoch when the first convergence has been completed after 5400 epochs, so as to evaluate the rapid re-initialization performance of PPP with the proposed method. The simulated cycle slip values of carrier phase observations on B1, B2, and B3 frequency are shown in Figure 2. Taking C01 as example, which represents the pseudo-random noise (PRN) of satellite 1 for Chinese BDS, the simulated cycle slip values are 4, −4, and 1 in cycles on B1, B2, and B3, respectively. The number of available satellite with triple frequency signals is always 8 during the test, as shown in Figure 2.

**Figure 3:**
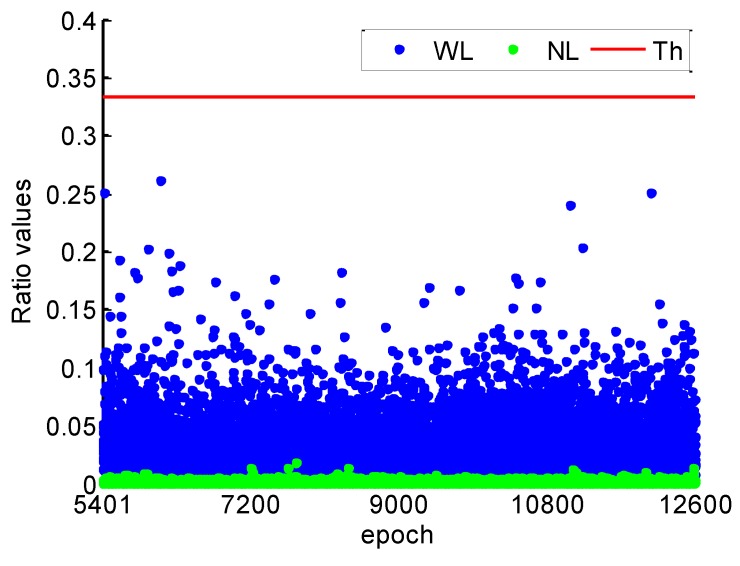
WL and NL ratio values for 1 s.

The ratio test values using LAMBDA for the WL and the NL are given in Figure 3. WL, NL, and Th in the caption of Figure 3 denote ratio test values of WL, NL, and threshold, respectively. It shows that all the ratio test values are much smaller than the threshold of 1/3, which illustrates the reliability of WL and NL cycle slips fixing with a high confidence level. The ratio values of NL are smaller than the WL, because the NL with TRIM has smaller ratio of noise to the wavelength and more observation redundancy than the WL. Furthermore, compared the simulated and the estimated cycle slip values, the success rate of cycle slip fixing achieves 100%.

**Figure 4:**
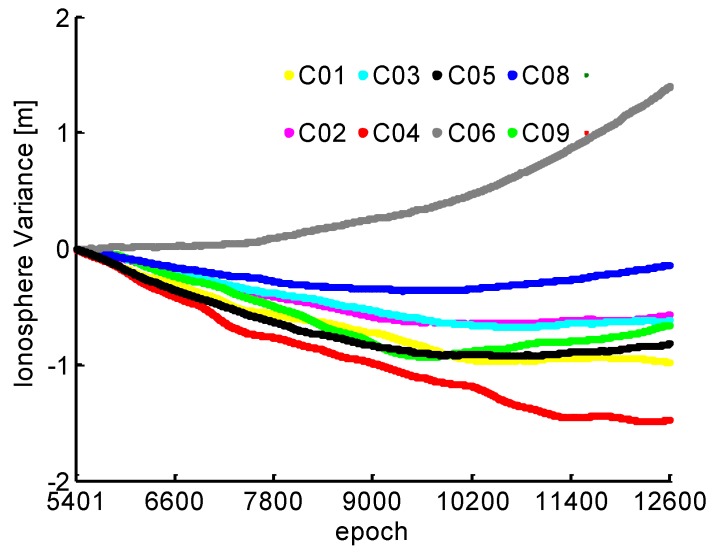
Relative ionospheric delay variation values.

**Figure 5:**
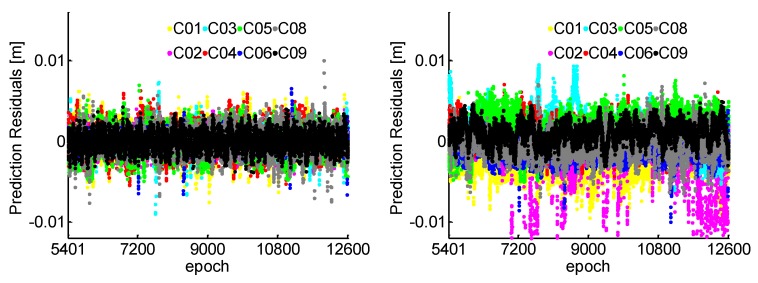
Ionospheric prediction residuals with 1 s interval. The left panel shows the residuals based on B1 and B2, the right panel based on B1 and B3.

For the NL cycle slip fixing, the estimated relative ionospheric delay variation based on the TRIM with 5 min during the injection of the simulation cycle slip in Figure 4. It can be found that the temporal correlation exists in the ionospheric delay between adjacent epochs. The prediction residuals for 1 s gap using the established TRIM are also plotted in Figure 5. We can see that millimeter accuracy can be obtained, which means the ionospheric variation can be precisely predicted for 1 s.

**Figure 6:**
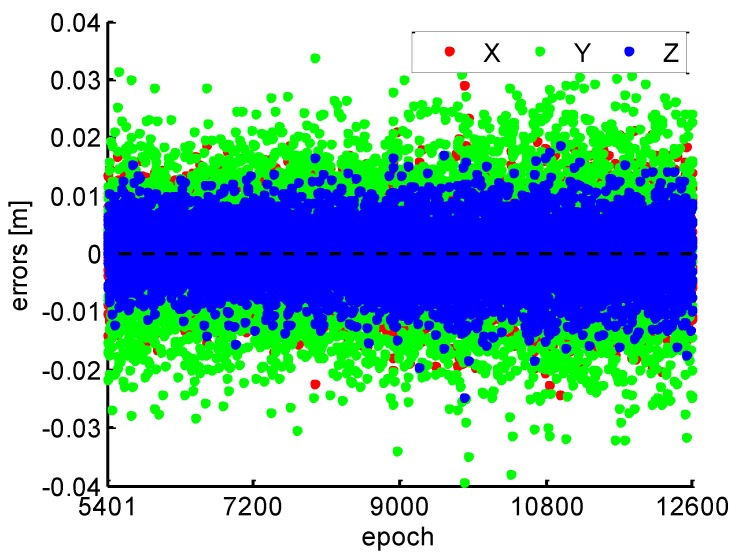
Relative position errors.

The relative position between adjacent epochs can be obtained after the NL cycle slip fixing successfully. Since the receiver antenna is static, the truth-value of relative position is zero, which is the perfect reference to assess the accuracy of estimated relative position. The relative position accuracy is at the order of centimeter level, as shown in Figure 6. X, Y, and Z in the caption of Figure 6 indicate position errors in the x, y, and z components of WGS-84 coordinate system. The results further verified the correctness and reliability of cycle slips fixing. Moreover, the position errors of PPP based on the proposed method can be convergence as Figure 1.

In order to test the performance of cycle slips fixing for the data gap with different time interval, the static data with 1 s interval are artificially decimated to generate 30 s, 60 s, 120 s, 180 s and 240 s, respectively. The static data with 1 s interval was introduced simulated cycle slips, as shown in Figure 2. The success rates of EWL, WL, and NL cycle slip fixing with different intervals are shown in Table 1. It has shown that the success rates of proposed cycle slip fixing method can also achieve 100% at each step even when the data gap reaches 180 s, which effectively mitigates the impact of ionospheric variation for cycle slip fixing with the data gap increasing. However, when the data gap reaches 240 s, although the WL cycle slip fixing success rate reaches 100%, but the NL drops to 87.5%.

**Figure 7:**
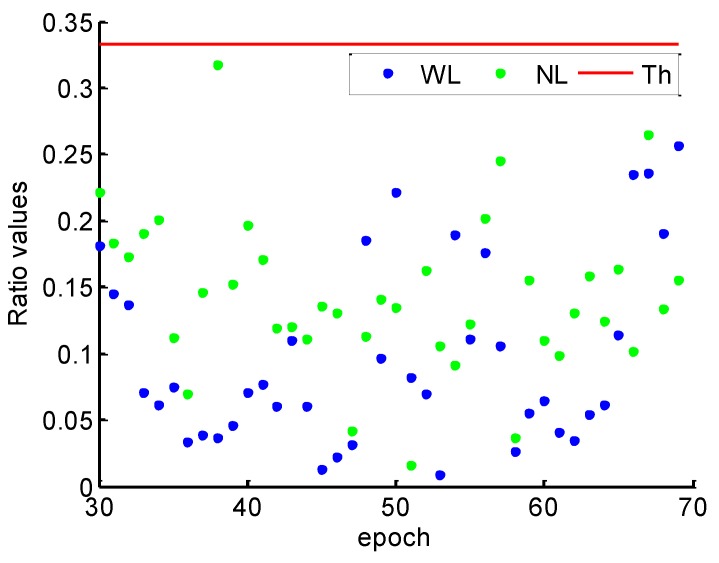
WL and NL ratio values for 180 s.

**Figure 8:**
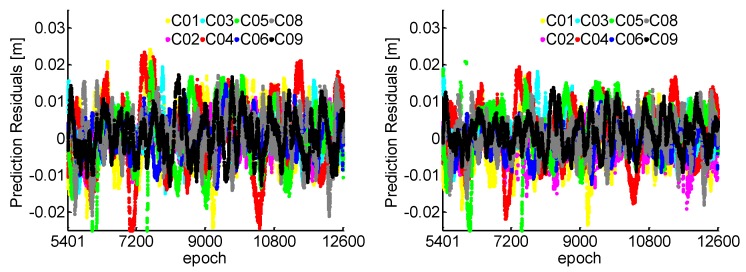
Ionospheric prediction residuals with 180 s intervals. The left panel shows the residuals based on B1 and B2, the right panel based on B1 and B3.

Figure 7 shows that all the ratio test values are smaller than 1/3 for 180 s data gap. The ratios of WL and NL are generally smaller for the 1 s interval than 180 s because the observation errors are strongly correlated in such a short span. Combined with Figure 3 and Figure 7, it can be found that the ratio values of NL increase more than the WL, because the NL with shorter wavelength is more susceptible to observation residuals than the WL. For the NL cycle slip fixing, the ionospheric prediction residuals for a 180 s interval using the TRIM are plotted in Figure 8. Compared with the 1 s interval as shown in Figure 5, the prediction accuracy of NL relative ionospheric based on the TRIM is slightly worse when the data gap increases.

**Figure 9:**
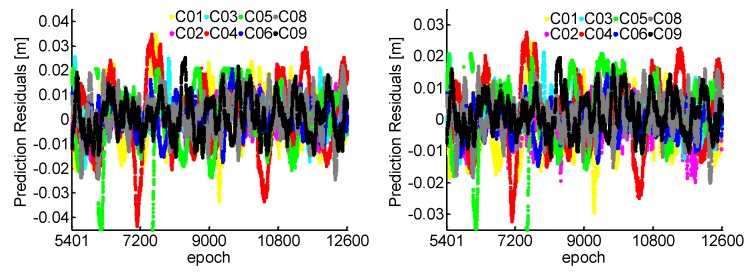
Ionospheric prediction residuals with 240 s intervals. The left panel shows the residuals based on B1 and B2, the right panel based on B1 and B3.

Table 2 shows the RMS of ionospheric prediction residuals based on TRIM in different intervals. The RMS of ionospheric variation prediction residuals increases with the increasing data gap, which causes the NL cycle slip fixing success rate drops to 87.5% when the data gap reaches 240 s. The ionospheric prediction residuals in 240 s intervals are plotted in Figure 9. Therefore, the success rate of NL cycle slip fixing will be affected by the ionospheric variation prediction accuracy based on TRIM. The WL cycle slip fixing based on the IF combination can effectively mitigate ionospheric delay.

**Figure 10:**
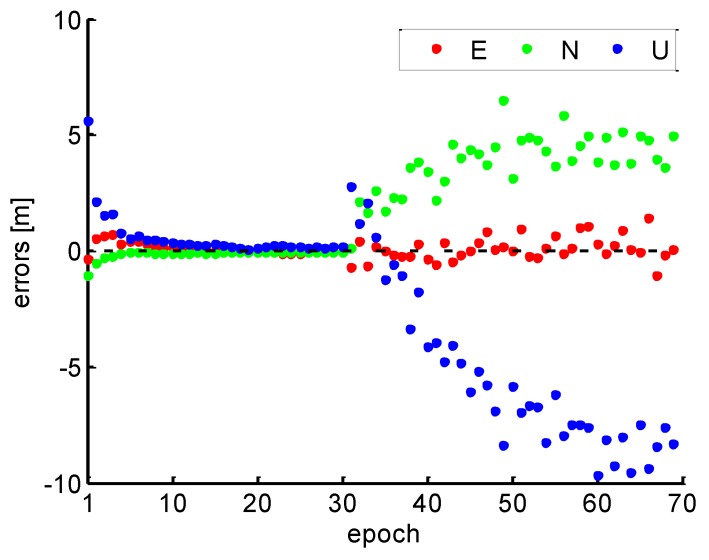
Conventional PPP solutions in 180 s intervals.

Based on the data which was introduced simulated cycle slips with 180 s interval, the position errors of the conventional PPP without cycle slip fixing are shown in Figure 10. It can be found that the position errors are divergent, and the continuous high precision of PPP is interrupted.

**Figure 11:**
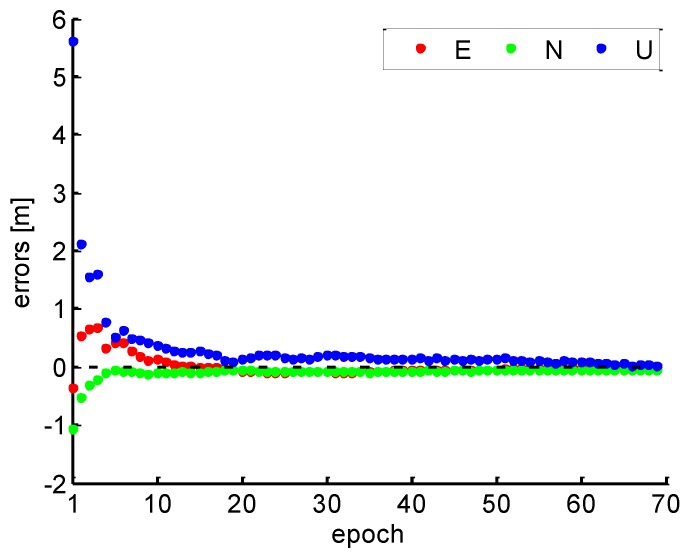
PPP solutions with cycle slip fixing in 180 s intervals.

In order to evaluate the performance of PPP rapid re-initialization and the validation of data gap based on the EWL-WL-NL cycle slips fixing method, the improved PPP solutions are given in Figure 11 by using the proposed cycle slips fixing method. Compared with the Figure 10, it is observed that the divergence position errors caused by cycle slips have been avoided and instantaneous re-initialization is achieved, which illustrates the correctness of cycle slips fixing and maintains continuous high precision positioning. Moreover, the instantaneous re-initialization can also be reproduced when the data gap is less than 180 s.

### 3.2. Kinematic Experiment

**Figure 12:**
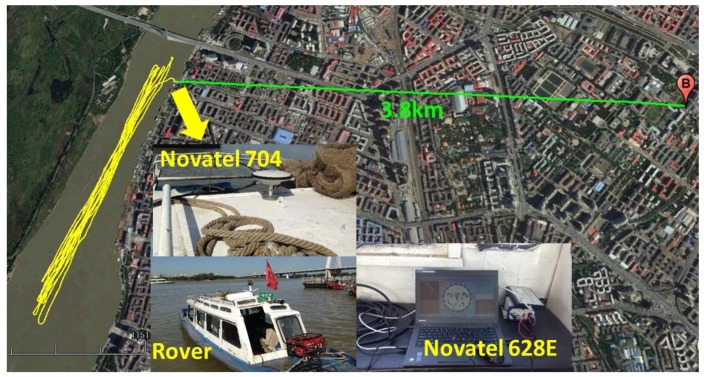
Kinematic experiment trajectory.

In order to further verify the effectiveness of the proposed method in the real-world applications, we use the real kinematic data with 1 s sampling interval collected by a Novatel 628E receiver and a 704 antenna on Songhua River of Harbin, China, at DOY 266, 2017. Figure 12 shows the traces of the kinematic experiment, the yellow line represents the trajectory of the boat, and the green line indicates the distance between the base station (the red symbol B) and the start point of rover. The kinematic PPP solutions were carried out to be compared by the RTK solutions for further verification. The *Kp* index for the ionosphere activity level is 2, which means a quiet ionosphere condition during the test.

**Figure 13:**
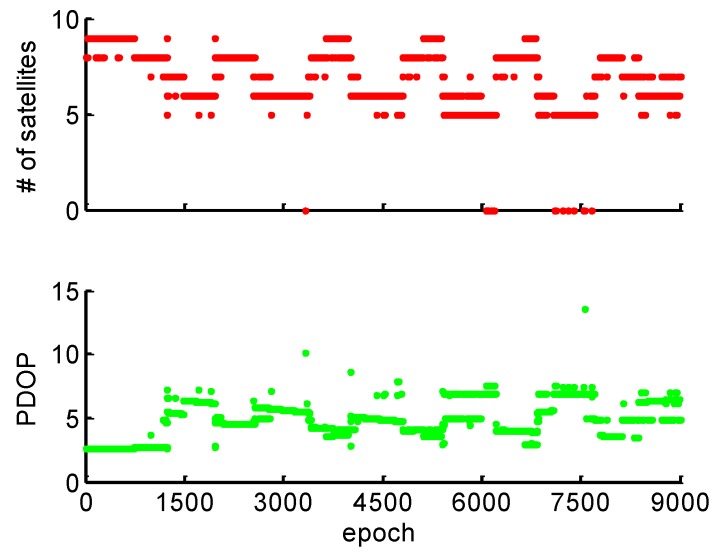
Available satellites and PDOP during the kinematic test.

Figure 13 shows the number of available satellites and the PDOP during the kinematic experiment. It can be observed that the number of available satellite changes frequently for the kinematic experiment. The PDOP is always larger than six when the available satellite number is less than seven. Because of frequent loss-of-lock in signal tracking, the available satellites changed more frequency under the challenging kinematic experimental environment, when compared with the simulated kinematic experiment.

**Figure 14:**
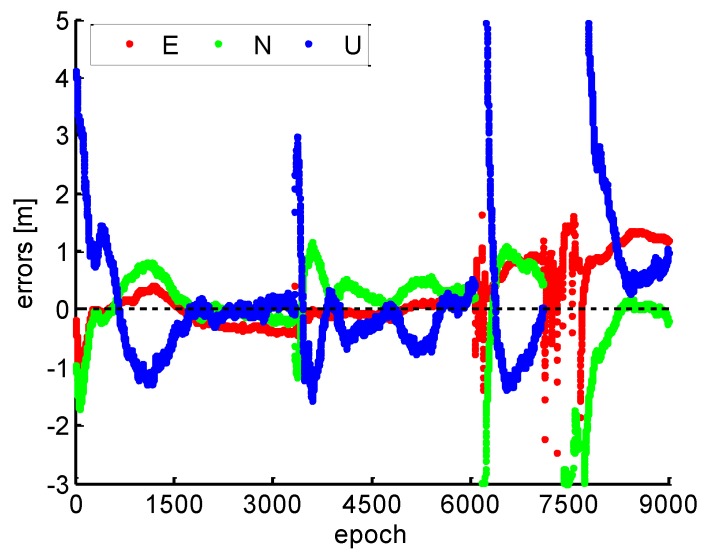
Position errors by the conventional PPP.

Figure 14 shows the position errors by the conventional PPP without cycle slip fixing. Combined with Figure 13, it can be found that the PPP will be re-initialized frequently and a long convergence is observed without cycle slip fixing when the available satellite number drops to zero, which limit the continuous high accuracy positioning performance of kinematic PPP.

**Figure 15:**
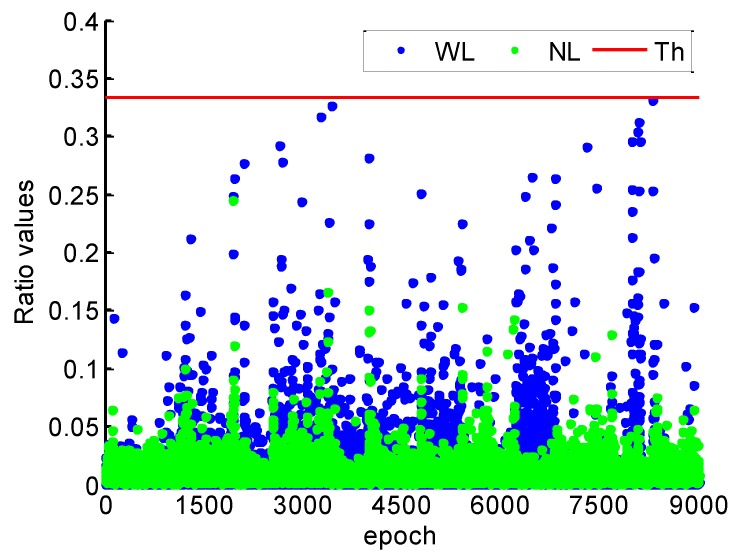
Ratio values of WL and NL.

Figure 15 shows the ratio values of WL and NL cycle slip fixing by the proposed method throughout the kinematic experiment. It can be found that the ratio values of WL and NL are smaller than 1/3, which illustrates the high reliability of cycle slips fixing. Compared with the ratio values of static data in Figure 3, the ratio values are much larger. It can be explained that the model strength of cycle slip fixing reduces by the severe multipath and few available satellite during the kinematic experiment.

**Figure 16:**
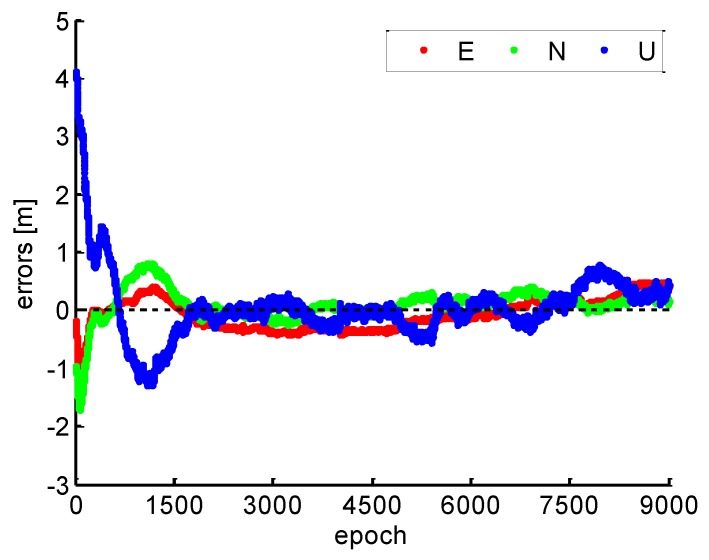
Position errors with the proposed cycle slip fixing method.

It can be found that the continuous high accuracy PPP can be obtained when the instantaneous re-initialization is achieved, as shown in Figure 16. Therefore, the precision of all carrier phase observation involved in positioning can be maintained without ambiguity parameters re-initialize when the cycle slips were fixed. It shows that the position errors in each positioning component by PPP with the proposed cycle slip-fixing method. After 30 min, it can be found that the position errors are less than 0.3 m, 0.1 m and 0.2 m in the east, north and vertical components, respectively.

## 4. Conclusions

The purpose of this study is to achieve the rapid re-initialization of PPP by fixing undifferenced cycle slip reliably. Hence, we propose a cascading EWL-WL-NL ionosphere-constrained cycle slip fixing method. Benefit from triple frequency observation combination, the epoch-differenced EWL and WL combinations under the IF criterion are constructed to achieve reliable cycle slip fixing, and the epoch-differenced NL combination mitigates ionospheric delay by the TRIM. Moreover, the strength of NL combination is also enhanced by the noise restricted and sufficient observation redundancy, which is beneficial for improving the reliability of cycle slip fixing. The proposed method is tested using static and kinematic data. The simulation kinematic experiment based on static data has shown that the ionospheric delay can be mitigated under a moderate ionosphere activity condition even though the data gap is up to several minutes, thus, the cycle slips can be reliably fixed with a high level of confidence when the triple-frequency carrier phase observations of all available satellites were introduced cycle slips. Moreover, the instantaneous re-initialization can be achieved after the first convergence. Furthermore, the long re-initialization time caused by loss-of-lock in signal tracking was removed, and the continuous sub-decimeter level PPP solutions can be maintained after the first convergence in the kinematic experiment. Therefore, applying the proposed cycle slip fixing method, the instantaneous re-initialization of PPP can always be achieved to provide continuous high accuracy position solutions.

Although the proposed method is capable of mitigating the ionospheric delay, the combined observation noise is another critical for cycle slip fixing. Therefore, the research of cycle slip fixing by mitigating ionospheric delay and restricting the combined observation noise simultaneously will be our future work. In addition, the performance of BDS triple-frequency cycle slip fixing is expected to be further improved when more accurate orbit and clock products and more BDS satellites redundancy are available in the future.

## Figures and Tables

**Table 1:** Cycle slip fixing success rate in different data gaps.

	Interval	30 s	60 s	120 s	180 s	240 s
Success Rate						
WL		100%	100%	100%	100%	100%
NL		100%	100%	100%	100%	87.5%

**Table 2:** RMS of prediction residuals based on TRIM in different data gaps.

	Interval	30 s	60 s	120 s	180 s	240 s
RMS						
B1/B2		0.002 m	0.003 m	0.005 m	0.007 m	0.009 m
B1/B3		0.002 m	0.003 m	0.004 m	0.006 m	0.008 m

## References

[1] Zumberge J.F., Heflin M.B., Jefferson D.C., Watkins M.M., Webb F.H. (1997). Precise point positioning for the efficient and robust analysis of GPS data from large networks. J. Geophys. Res. Solid Earth.

[2] Yang F.X., Zhao L., Li L., Feng S.J., Cheng J.H. (2018). Performance evaluation of kinematic BDS/GNSS real-time precise point positioning for maritime positioning. J. Navig..

[3] Seepersad G., Bisnath S. (2015). Reduction of PPP convergence period through pseudorange multipath and noise mitigation. GPS Solut..

[4] Banville S., Langley R.B. Improving real-time kinematic PPP with instantaneous cycle-slip correction. Proceedings of the 22nd International Technical Meeting of the Satellite Division of the Institute of Navigation (ION GNSS 2009).

[5] Astafyeva E., Yasyukevich Y., Maksikov A., Zhivetiev I. (2014). Geomagnetic storms, super-storms, and their impacts on GPS-based navigation systems. Space Weather.

[6] Kintner P., Ledvina B., de Paula E. (2007). GPS and ionospheric scintillations. Space Weather.

[7] Blewitt G. (2013). An automatic editing algorithm for GPS data. Geophys. Res. Lett..

[8] Cai C.S., Liu Z.Z., Xia P.F., Dai W.J. (2013). Cycle slip detection and repair for undifferenced GPS observations under high ionospheric activity. GPS Solut..

[9] Liu Z.Z. (2011). A new automated cycle slip detection and repair method for a single dual-frequency GPS receiver. J. Geodesy.

[10] Guo F., Zhang X., Wang J., Ren X. (2016). Modeling and assessment of triple-frequency BDS precise point positioning. J. Geodesy.

[11] Dai Z., Knedlik S., Loffeld O. (2009). Instantaneous triple-frequency GPS cycle slip detection and repair. Int. J. Navig. Obs..

[12] Lacy M.C.D., Reguzzoni M., Fernando S. (2012). Real-time cycle slip detection in triple-frequency GNSS. GPS Solut..

[13] Zhao Q.L., Sun B.Z., Dai Z.Q., Hu Z.G., Shi C., Liu J.N. (2015). Real-time detection and repair of cycle slips in triple-frequency GNSS measurements. GPS Solut..

[14] Huang L.Y., Lu Z.P., Zhai G.J., Ouyang Y.Z., Huang M.T., Lu X.P., Wu T.Q., Li K.F. (2015). A new triple-frequency cycle slip detecting algorithm validated with BDS data. GPS Solut..

[15] Bisnath S.B. Efficient, Automated Cycle-Slip Correction of Dual-Frequency Kinematic GPS Data. Proceedings of the 13th International Technical Meeting of the Satellite Division of the Institute of Navigation (ION GPS 2000).

[16] Banville S., Langley R.B. (2013). Mitigating the impact of ionospheric cycle slips in GNSS observations. J. Geodesy.

[17] Zhang X.H., Li X.X. (2012). Instantaneous re-initialization in real-time kinematic PPP with cycle slip fixing. GPS Solut..

[18] Zhang X.H., Li P. (2016). Benefits of the third frequency signal on cycle slip correction. GPS Solut..

[19] Remondi B.W. (1985). Global Positioning System carrier phase: Description and use. J. Geodesy.

[20] Li L., Jia C., Zhao L., Yang F.X., Li Z.S. (2017). Integrity monitoring-based ambiguity validation for triple-carrier ambiguity resolution. GPS Solut..

[21] Xiao G.R., Mayer M., Heck B., Sui L.F., Zeng T., Zhao D.M. (2018). Improved time-differenced cycle slip detect and repair for GNSS undifferenced observations. GPS Solut..

[22] Kouba J. (2009). A Guide to Using International GNSS Service (IGS) Products. https://igscb.jpl.nasa.gov/igscb/resource/pubs/UsingIGSProductsVer21.pdf.

[23] Feng Y.M. (2008). GNSS three carrier ambiguity resolution using ionosphere-reduced virtual signals. J. Geodesy.

[24] Hatch R. The synergism of GPS code and carrier measurements. Proceedings of the Third International Symposium on Satellite Doppler Positioning at Physical Sciences Laboratory of New Mexico State University.

[25] Melbourne W.G. The case for ranging in GPS-based geodetic systems. Proceedings of the First International Symposium on Precise Positioning with the Global Positioning System.

[26] Wubbena G. Software developments for geodetic positioning with GPS using TI-4100 code and carrier measurements. Proceedings of the First International Symposium on Precise Positioning with the Global Positioning System.

[27] Teunissen P.J.G. (1995). The least squares ambiguity decorrelation adjustment: A method for fast GPS integer estimation. J. Geodesy.

[28] Dai L., Wang J., Rizos C., Han S. (2003). Predicting atmospheric biases for realtime ambiguity resolution in GPS/GLONASS reference station networks. J. Geodesy.

[29] Geng J., Meng X., Dodson A.H., Ge M., Teferle F.N. (2010). Rapid re-convergences to ambiguity-fixed solutions in precise point positioning. J. Geodesy.

[30] Montenbruck O., Steigenberger P., Khachikyan R., Weber G., Langley R.B., Mervart L., Hugentobler U. (2014). IGS-MGEX: Preparing the ground for multi-constellation GNSS science. Inside GNSS.

[31] http://www.swpc.noaa.gov/products/planetary-k-index.

